# Review of a frugal cooling mattress to induce therapeutic hypothermia for treatment of hypoxic-ischaemic encephalopathy in the UK NHS

**DOI:** 10.1186/s12992-022-00833-5

**Published:** 2022-04-21

**Authors:** Giulia Dallera, Mark Skopec, Cheryl Battersby, James Barlow, Matthew Harris

**Affiliations:** 1 Department of Primary Care and Public Health, Imperial College London, London, UK; 2 Consultant Neonatologist, Chelsea and Westminster Hospital NHS Foundation Trust, London, UK; 3 Imperial College Business School, Imperial College London, London, UK

**Keywords:** Frugal innovation, Reverse innovation, Therapeutic hypothermia, Hypoxic ischaemic encephalopathy, Phase change materials

## Abstract

Hypoxic ischaemic encephalopathy (HIE) is a major cause of neonatal mortality and disability in the United Kingdom (UK) and has significant human and financial costs. Therapeutic hypothermia (TH), which consists of cooling down the newborn’s body temperature, is the current standard of treatment for moderate or severe cases of HIE. Timely initiation of treatment is critical to reduce risk of mortality and disability associated with HIE. Very expensive servo-controlled devices are currently used in high-income settings to induce TH, whereas low-income settings rely on the use of low-tech devices such as water bottles, ice packs or fans. Cooling mattresses made with phase change materials (PCMs) were recently developed as a safe, efficient, and affordable alternative to induce TH in low-income settings. This frugal innovation has the potential to become a reverse innovation for the National Health Service (NHS) by providing a simple, efficient, and cost-saving solution to initiate TH in geographically remote areas of the UK where cooling equipment might not be readily available, ensuring timely initiation of treatment while waiting for neonatal transport to the nearest cooling centre. The adoption of PCM cooling mattresses by the NHS may reduce geographical disparity in the availability of treatment for HIE in the UK, and it could benefit from improvements in coordination across all levels of neonatal care given challenges currently experienced by the NHS in terms of constraints on funding and shortage of staff. Trials evaluating the effectiveness and safety of PCM cooling mattresses in the NHS context are needed in support of the adoption of this frugal innovation. These findings may be relevant to other high-income settings that experience challenges with the provision of TH in geographically remote areas. The use of promising frugal innovations such as PCM cooling mattresses in high-income settings may also contribute to challenge the dominant narrative that often favours innovation from North America and Western Europe, and consequently fight bias against research and development from low-income settings, promoting a more equitable global innovation landscape.

## Introduction

The need to solve problems in resource-constrained settings often leads to the development of simple but very effective solutions. These frugal innovations help to increase access to healthcare in contexts where treatments are unaffordable or unavailable [1]. However, they often have the potential to become reverse innovations and improve healthcare in high-income countries (HICs), by saving costs to health systems and increasing efficiency [2, 3]. Cooling mattresses made with phase change materials (PCMs) to induce therapeutic hypothermia (TH) for the treatment of neonatal hypoxic ischaemic encephalopathy (HIE) are one such example. They are a promising and affordable alternative to initiate treatment of HIE in remote areas of HICs, where expensive servo-controlled cooling devices or transport to the nearest cooling centre might not be readily available, often delaying start of treatment and increasing risk for mortality or disability. The National Health Service (NHS) in the United Kingdom (UK) experiences several challenges in the provision of TH, including the increasing number of neonates in need of intensive care, geographical disparity in the availability of cooling equipment, and the need to rapidly transport neonates born in non-cooling centres to the nearest intensive care unit. Based on the findings from a comprehensive narrative review of the literature, this paper critically evaluates the suitability of PCM cooling mattresses for use by the NHS in the UK and demonstrates that there is a clinical and economic case for their adoption in remote or rural areas of the country. These findings may be generalisable to other HICs that experience geographical disparity in the availability of treatment for HIE, because of lack of access to cooling equipment in geographically remote areas.

## Methods

We conducted a comprehensive narrative review of the literature on the use of PCM cooling mattresses to induce TH for the treatment of HIE, their effectiveness in comparison with existing alternatives, and on current practices and challenges in relation to treatment of HIE in the UK. This consisted in doing extensive research of mainly grey literature sources from Web of Science, Scopus and Google Scholar, and adopting a snowballing approach. In this review, the term “high-tech cooling devices” is used to indicate expensive and technologically advanced equipment for TH commonly used in HICs such as servo-controlled automated cooling devices (e.g. Blanketrol II, Tecotherm Neo, Tecotherm HELIX, Tecotherm TS 200), while the term “low-tech cooling devices” refers to less sophisticated technologies that are more commonly used to induce TH in LMICs, such as ice packs, fans, or water bottles.

## Background

### Hypoxic ischaemic encephalopathy

HIE is a severe neonatal brain injury that occurs as a consequence of anoxia, or lack of oxygen in the brain during the neonatal period, preventing adequate blood flow to the infant’s brain [4]. HIE is a series of physiological, cellular and molecular events that lead to neuronal death within hours or days from the initial injury and can cause premature death or life-long disability. HIE consists of two energy failure phases where neuronal loss occurs, but that are separated by a six-hour latent phase where cerebral circulation is restored to normal [5]. This latent phase has been identified as a therapeutic window to delay the neuronal death associated with the second energy failure phase, and thereby reduce the risk of mortality and disability [5–8]. Infants affected by HIE may survive with acute conditions such as seizures, altered states of consciousness, breathing difficulties, weak muscle tone and metabolic disorders, or chronic conditions such as cerebral palsy, epilepsy and other cognitive and behavioural problems [9].

HIE affects 10-20 in 1000 neonates in low- and middle-income countries (LMICs), and 1.5 in 1000 neonates in HICs [10]. In the UK, HIE is a major cause of mortality and disability in near-term and term neonates [11]. It affects 2.96 out of every 1000 live births and makes up 3% of neonatal unit admissions [12]. About 1000 neonates die of HIE in the UK every year [9, 13]. HIE causes significant financial and human costs to newborns and their families, healthcare professionals and the wider society. In 2000, it was estimated that preventing 10% of birth-related adverse events leading to brain injury could save the NHS about £20 million every year [14]. The UK government has committed to halve brain injuries at birth by 2030, as part of a larger ambition to reduce stillbirths as well as neonatal and maternal death [15]. This is in line with Sustainable Development Goal 3.2, which aims to reduce neonatal mortality to at least 12 out of every 1000 births in every country by 2030 [16].

### Therapeutic hypothermia as a treatment for hypoxic ischaemic encephalopathy

TH is the current standard of treatment for moderate or severe cases of HIE in term and near-term infants born 36 weeks or greater [17, 18]. It consists of cooling down the newborn’s body to 33.5 °C for 72 h starting within 6 h of birth, targeting the therapeutic window between the primary and secondary energy failure phases [19]. TH works by reducing metabolic activity, consequently decreasing the brain’s oxygen requirement, and mitigating neuronal injury and death. The neural protective effect of TH is multifactorial, including suppression of inflammation, intracellular signalling and programmed neuronal death [5]. Although TH does not prevent mortality and disability altogether, it is effective in reducing risk of death and brain damage in infants with moderate to severe HIE by approximately 12% at 18 months of age [19, 20].

TH can be achieved either through selective head cooling with caps, or wholebody cooling with blankets, mattresses or ice packs, or both. The rationale behind selective head cooling is that 70% of total body heat is produced by the infant’s brain, as well as to prevent any damage that systemic hypothermia would cause to the infant’s body [18, 21]. To date, there is no evidence to suggest that one technique is better than the other [22, 23]. Very expensive servo-controlled devices are used to induce TH in HICs whereas low-technology alternatives (such as ice packs, fans or water bottles) have been used in low-income countries (LICs). The cost of servo-controlled devices used in HICs ranges between £20,000 and £30,000 [24]. Relatively recently, mattresses made with PCMs have been developed as a reliable and significantly more affordable method for TH in low-resource settings, as they cost about one tenth of high-tech devices used in HICs [25, 26]. Most research on the use of PCM mattresses has been conducted in LICs, where they offer substantial improvement compared to other low-tech cooling devices such as ice packs, fans or water bottles in terms of effectiveness, temperature control, and safety [27, 28].

## Uses and applications of phase change materials

PCMs store and release thermal energy at a specific temperature while passively transitioning between liquid and solid phases, without the need for constant electrical supply or water. PCMs are usually made of salt hydride, fatty acid and esters or paraffin and have a variety of applications. They are used by firefighters, athletes or surgeons to stabilise temperature fluctuations, and are a promising application in transport, for example to replace refrigerated trucks for the delivery of food, as well as in housing, to avoid the gain or loss of heat in buildings through walls [29–31]. PCMs are solid at room temperature but act as heat sinks, making them suitable to induce TH. When a newborn is in contact with PCMs, these absorb body heat until the melting temperature is reached, leading to phase change. This causes reduction in the newborn’s body temperature for an extended period of time. PCMs in the liquid phase can re-solidify by releasing heat over a period of between six-eight hours. When not in use, they are stored in refrigerators.

Researchers from the Karolinska Institute in Stockholm, Sweden, were the first to explore the use of PCMs to induce TH on piglets in 2007, showing that these were an easy and effective alternative to water bottles to reach and sustain a target temperature [29, 32]. The first human trial using PCMs was conducted at Calicut Medical College in Kerala, India, in 2009 [30]. A medical device using a PCM cascaded system to induce TH in neonates suffering from HIE was commercialised in India in 2014 under the name of MiraCradle, and subsequently patented [33–36]. PCM mattresses are a frugal innovation, meeting six of the ten core competencies for successful development of frugal solutions towards the achievement of global sustainability [37] (Table 1).

**Table 1:** Core competencies for successful frugal innovation development met by PCM cooling mattresses [33, 37]

Feature of frugality	Description
Ruggedisation	• Designed to be used in resource-poor settings. • Made with tough and long-lasting material. Can be repetitively used for over three years. • No requirement of constant electricity input, other than a fridge to pre-cool PCMs.
Lightweight	• Portable given the size (620 mm × 460 mm) and weight (13 kg) of the device. • Ideal for transportation.
Human centric design	• Intuitive and easy to use, with no requirement of additional training. • Comfortable for babies.
Simplification	• Made of essential components to induce TH compared with high-tech servo-controlled cooling devices.
Adaptation	• An extra PCM layer with lower melting temperature can be added to the device to aid cooling and stabilize temperature in environments with high external temperature.
Affordability	• Cost is 1/10th of high-tech cooling devices (about £1800). • No recurring expenses or maintenance costs.

## Components and key features of PCM cooling mattresses

**Fig. 1:**
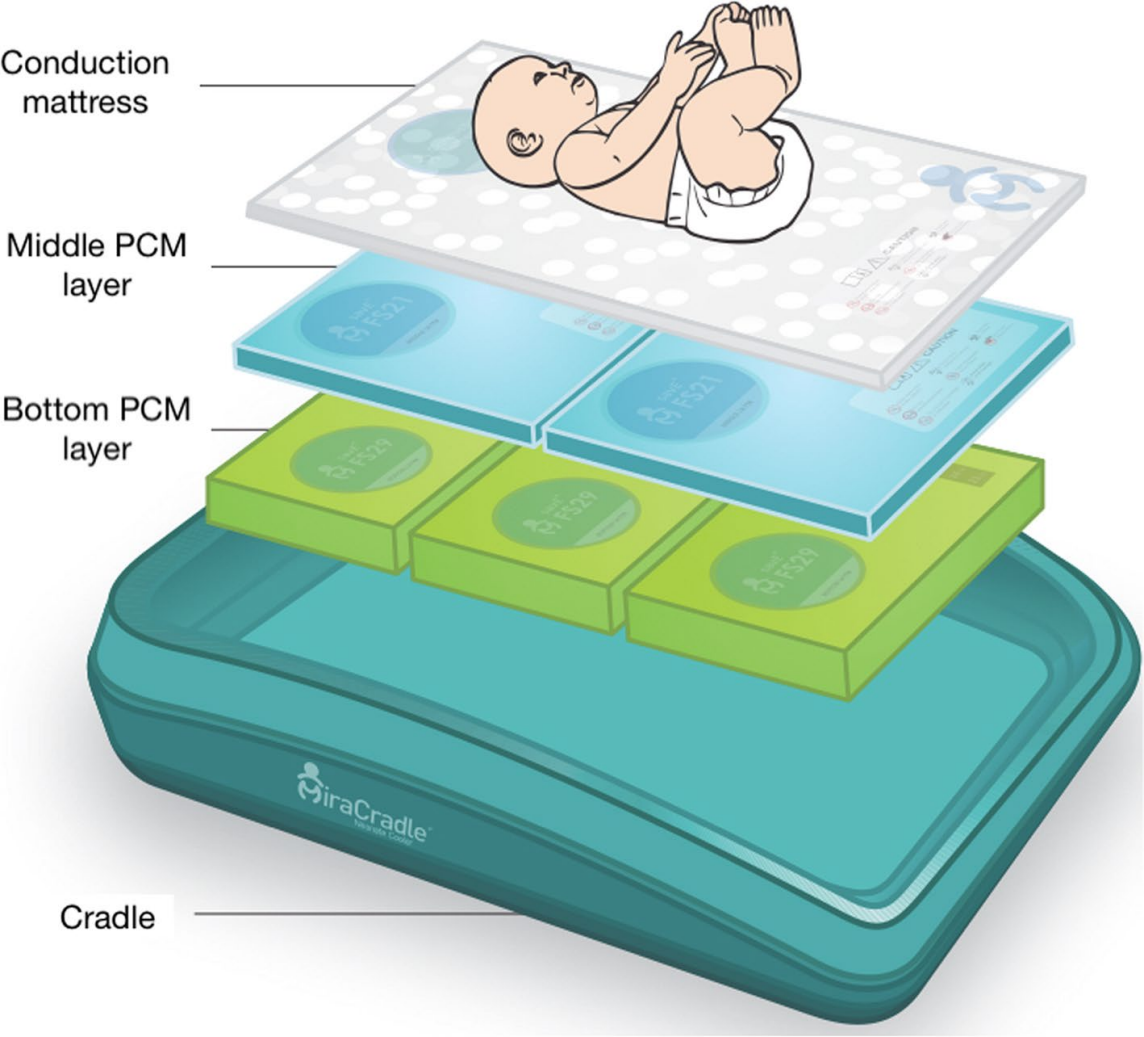
Cooling mattress with a PCM cascaded system to induce TH in neonates suffering from HIE [33]. Reproduced with permission from the manufacturer

Cooling mattresses that employ a PCM cascaded system to induce TH are made of four components: an insulated cradle, two PCM layers, each with specific melting and freezing temperatures, thicknesses and conductivities, and a conduction mattress, as shown in Fig. 1 [33]. The cascaded system is achieved by using different layers of PCMs that melt or freeze at specific temperatures.

Each component is described in greater detail in Table 2.

**Table 2:** Key components of PCM cooling mattresses for the induction of TH [33]

Component	Description
Insulated cradle	• Provides the base for the other components. • Made of plastic. • Insulated to make temperature control last longer.
Bottom PCM layer	• Melting point of 29.0 °C. • Core component of the PCM cooling mattress. • Helps to achieve therapeutic hypothermia by passively absorbing the heat from the newborn’s body.
Middle PCM layer	• Melting point of 21 °C. • Can be added during induction of therapeutic hypothermia in case the bottom PCM layer is not sufficient to achieve target temperature of 33.5 °C, for example due to high temperature of the external environment.
Conduction mattress	• Made of gel. • Helps to transfer the heat from the newborn’s body to the PCM layers. • Provides a smooth surface for the baby to lie on.

**Fig. 2:**
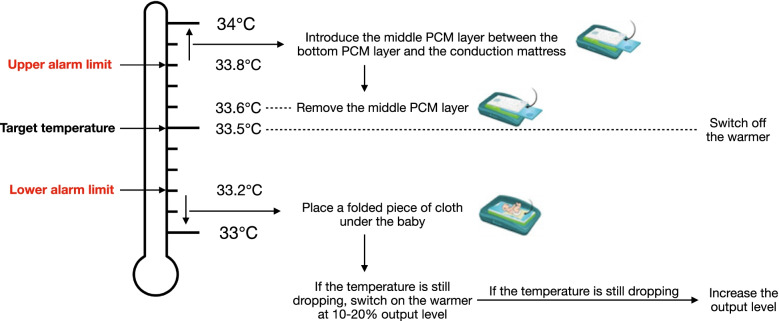
Steps for the induction of TH using PCM cooling mattresses are illustrated [33]. Reproduced with permission from the manufacturer

This PCM cascaded system creates a cooling device that operates as if it was quasi-automated by passively absorbing and releasing heat to maintain the target temperature. Precise temperature control of 33-34.5 °C can be maintained for 72 h, while requiring minimal staff input and no continuous electrical supply other than the fridge required to pre-cool the PCMs [33]. PCM layers are enclosed with a polymer matrix that prevents risk of leakage between phase changes. PCM cooling mattresses are used in conjunction with respiratory and circulatory monitoring equipment, a rectal probe to monitor vital parameters, a refrigerator to pre-cool PCMs, and a warmer both to aid the rewarming phase and to adjust body temperature in case it drops below 32 °C, although this is not common and may occur in less than 10% of cases [38] (Fig. 2).

## Comparison with existing technologies and evidence for effectiveness

PCM cooling mattresses make use of entirely new materials and components compared to low- and high-tech devices that are currently used to induce TH. Table 3 details their relative advantage compared to high-tech and low-tech solutions for inducing TH.

**Table 3:** PCM cooling mattresses offer relative advantage compared to existing cooling devices

Feature	PCM cooling mattresses	High-tech cooling devices	Low-tech cooling devices
Cooling induction time	**Rapid**	Rapid	Slow
Temperature fluctuations	**No**	No	Yes
Slow and controlled rewarming	**Slow and controlled**	Slow and controlled	Hard to control
Cost	**~£1800**	~£20,000-30,000	Low cost but higher risks of complications
Portable	**Yes**	Not always	Yes
Recurring expenses	**No**	Yes (e.g. disposable cooling blankets for Blanketrol II)	Yes (e.g. ice packs)
Staff input for device monitoring	**Moderate**	Low	High
Training	**Low**	High	Low
Affected by environmental temperature	**No**	No	Yes
Risk of adverse events	**Minimal**	Minimal	High

The performance of PCM cooling mattresses is comparable to high-tech devices currently used in HICs in terms of TH induction time, maintenance of target temperature and controlled and slow rewarming, while also offering the advantage of being significantly cheaper, portable, and requiring minimal training for use [38–41]. Table 4 provides evidence for effectiveness of PCM cooling mattresses. Key studies or trials of existing cooling devices to induce TH, both low- and high-tech, have been summarised to compare their performance with that of PCM cooling mattresses.

**Table 4:** Evidence for effectiveness of PCM cooling mattresses in comparison with low- and high-tech cooling devices

Study	Cooling device	Sample	Country	Cooling induction time	Achieved temperature (mean ± SD °C)	Rewarming
ICE [42]	Ice packs	221 (110 cooled, 111 controls)	Australia, New Zealand, Canada, USA	2 h (IQR = 1-3 h)	33.8 ± 0.4 °C for 72 h	0.5 °C every two hours
Horn et al., 2009 [43]	Servo-controlled fan	10 (no controls)	South Africa	58 min	33.6 ± 0.2 °C for 72 h	0.2 °C every 30 min
Robertson et al., 2008 [44]	Water bottles	36 (21 cooled, 15 controls)	Uganda	1 h	33.62 ± 0.69 °C for 72 h	< 0.5 °C per hour
TOBY [40]	High-tech manual cooling device (Tecotherm TS 200)	325 (163 cooled, 162 controls)	UK	NA	33.5 ± 0.5 °C for 72 h	0.5 °C per hour
NICHD [39]	High-tech servo-controlled cooling device (Blanketrol II)	205 (102 cooled, 106 controls)	USA	90 min	33.4 ± 0.9 °C for 72 h	0.5 °C per hour
Oliveira et al., 2018 [45]	Low-cost servo-controlled cooling device (Tecotherm HELIX)	82 (no controls)	India	1.7 h (SD = 1.5)	33.4 ± 0.2 °C for 72 h	0.34 °C per hour
Thayyil et al., 2021 [46]	Low-cost servo-controlled cooling device (Tecotherm Neo)	408 (202 cooled, 206 controls)	India, Bangladesh, Sri Lanka	NA	33.5 ± 0.1 °C for 72 h	0.5 °C per hour
Thomas et al., 2015 [41]	PCM cooling mattress (MiraCradle)	41 (no controls)	India	30 min (IQR = 10-90 min)	33.45 ± 0.26 °C for 72 h	0.24 °C per hour
Thomas et al., 2017 [38]	PCM cooling mattress (MiraCradle)	103 (no controls)	India	90 min (IQR = 45-120 min)	33.5 ± 0.39 °C for 72 h	0.28 °C per hour
THIN [47]	PCM cooling mattress (MiraCradle)	50 (25 cooled, 25 controls)	India	NA	33.5 ± 0.27 °C for 72 h	0.2 °C-0.5 °C per hour
Catherine et al., 2021 [48]	PCM cooling mattress (MiraCradle)	172 (78 cooled, 84 controls)	India	NA	33.5 ± 0.5 °C for 72 h	0.5 °C per hour

A multi-centre study conducted in India reported lower risk of adverse events when using PCM cooling mattresses compared with evidence from servo-controlled devices, although it was not a randomised controlled trial and the number of infants with severe HIE was relatively low [38]. In a recent randomised controlled trial conducted in India, TH was successfully induced by PCM cooling mattresses in infants suffering from moderate to severe HIE [47]. This trial also demonstrated that TH induced by PCM cooling mattresses has neuroprotective effects on MRI biomarkers, although this was only evaluated on 22 infants so further evidence is required to substantiate these benefits. Another randomised controlled trial from India showed that induction of TH using a PCM cooling mattress in infants suffering from moderate to severe HIE was successful in reducing mortality and neurological defects at 18 months [48]. PCM cooling mattresses offer substantial improvement with respect to devices currently used in low-resource settings such as ice packs, cooling fans or water bottles, which have been associated with slower cooling times, greater temperature fluctuations, risk of shivering and necrosis of subcutaneous fat, and more frequent staff input since a set temperature cannot be controlled [27, 28, 42, 49].

Although PCM cooling mattresses perform similarly to high-tech servo-controlled devices used in HICs at about one tenth of the cost [50], they do require higher staff input because, unlike servo-controlled devices, they are not fully automated and the lack of close monitoring by nursing staff may increase risks of complications such as over-cooling, and consequently the need for intensive care [30, 51]. PCM cooling mattresses do not require maintenance, constant electricity (other than a refrigerator to pre-cool PCMs) or recurring expenses. A cost-effectiveness analysis comparing PCM cooling mattresses with servo-controlled devices may be required to evaluate whether the low cost of this frugal innovation is offset by the additional staff input it necessitates in terms of operating the device, as well as the amount of intensive care required, including respiratory and circulatory monitoring and management. Recently reported data from the HELIX trial using a high-tech servo-controlled device to induce TH in South Asia has suggested that TH actually increases mortality in LMICs and should therefore be avoided [46]. These findings call for further research into the effectiveness of cooling in these contexts, to evaluate the appropriateness of PCMs as an alternative to servo-controlled devices where neonatal therapeutic hypothermia is recommended, for example in the UK.

PCM devices are not the only low-cost alternatives to servo-controlled technology to induce TH. A low-cost servo-controlled device for whole-body cooling was recently tested in India and was shown to be effective in reaching and maintaining target temperature, offering a promising and affordable alternative to manual cooling devices currently used in LICs [45]. While reviewing the effectiveness of this low-cost servo-controlled cooling device to induce TH was outside the scope of this study given its similarity to expensive servo-controlled devices in terms of functioning and components, there is a strong need for randomised controlled trials that compare PCM cooling mattresses with both low- or high-tech devices to further test their effectiveness and safety.

### Adoption of PCM cooling mattresses by the UK NHS

#### Current practice of therapeutic hypothermia in the NHS and existing challenges

In the UK, active TH with intra-corporeal monitoring was recommended as a standard treatment for moderate and severe cases of HIE in 2010 [52, 53]. These guidelines reflect those published by the International Liaison Committee on Resuscitation in 2010 [54]. In the UK, guidance was provided by the TOBY Cooling Register, set up in 2006 to ensure uniformity of practice for TH and to monitor national uptake of the procedure [55]. It was estimated that TH as a treatment for HIE using servo-controlled cooling devices saves the NHS and families £200 million every year by reducing the number of children suffering from disability [56].

The NHS still experiences several challenges in the provision of TH. A recent survey highlighted significant disparity in the availability of cooling equipment at the national level, ranging from 21% in Yorkshire & Humber to 92% in Kent, Surrey & Sussex [57]. Out of the 57 neonatal intensive care units (NICUs) in the UK, only 2 are not equipped with cooling devices, while at least half of local neonatal units (LNUs) (86 in total) do not have cooling devices at all. Servo-controlled cooling devices are the current standard of treatment, however there is no evidence comparing the use of PCM cooling mattresses or manual cooling methods to servo-controlled devices in UK neonatal units [58]. Neonatal care in the UK is delivered by a network model, with a designated cooling centre in each network. This centralisation of care enables units to develop the expertise in the evaluation of infants suffering from HIE and their eligibility to TH, the use of the equipment, follow-up brain imaging and interpretation, to deliver the best clinical care. However, half of neonates with HIE in the UK are born in centres that are not equipped with cooling devices and thus require transport to the nearest neonatal unit [59]. While all neonatal transport teams are equipped with servo-controlled cooling devices, HIE is a time-critical condition and delays in the start of treatment increase the risk for mortality and disability [40, 57]. A recent study showed that neonates born in non-cooling centres are at greater risk of experiencing seizures upon admission to cooling centres compared to those born in hospitals equipped with cooling devices [59]. Although the reasons behind such disparity in treatment availability were not investigated in the study, the general recommendation made by the authors was to equip all levels of neonatal care with cooling devices. Special care baby units (SCBUs) and LNUs should at least be prepared to initiate TH and to monitor cerebral function while waiting for the arrival of transport teams to transfer neonates to the nearest NICU [57, 60]. Adding further complexity to the issue of availability of equipment is the subjectivity of criteria for eligibility to TH, and the need for expertise to correctly evaluate infants suffering from HIE [61].

#### Rationale for adoption

##### Improving current practice and reducing costs

Neonatal units in the UK are under pressure due to staff shortages, constraints on funding, and the increasing number of neonates in need of neonatal care [62]. Increasing availability of expensive servo-controlled cooling equipment nationally in all neonatal units is neither compatible with the centralised structure of neonatal care in the UK, nor sustainable given the cost of servo-controlled cooling devices and the expertise required to correctly evaluate infants suffering from HIE and their eligibility to TH, initiate and conduct cooling, monitor vital parameters and treat any side effects [17]. Whilst it is unlikely for PCM cooling mattresses to replace servo-controlled devices already available in the majority of NICUs, they may offer a low-cost, safe and more controlled alternative to passive cooling in SCBUs and LNUs while waiting for the arrival of neonatal transport teams, particularly in geographically remote areas where travel distances can be significant. In these contexts, ensuring timely initiation of TH whilst waiting for transport to a central NICU, could be a significant improvement on current care [27, 28, 42]. In the UK, provision of one servo-controlled cooling device for every SCBU (46 in total) and LNU (86 in total) would cost ~£3,300,000, while only ~£237,600 would be enough to equip *all* SCBUs and LNUs with at least one PCM cooling mattress. Paramedic services are already equipped with servo-controlled cooling devices, however initiation of TH using PCM cooling mattresses in SCBUs and LNUs may ease pressure on neonatal transport services, ensuring that neonates can reach hospitals within the target temperature range [63]. Neonatal units or transport services in the UK do not generally have electricity issues, however PCM cooling mattresses offer the opportunity of inducing TH sustainably given that they do not require constant electric supply. In fact, sustainability is a key feature of frugal innovations [37]. Although there is a strong case for expanded adoption of PCM cooling mattresses into SCBUs and LNUs in remote areas of the UK, it should be accompanied by improvements in coordination within networks across levels of neonatal care.

##### Regulatory environment and the need for evidence of effectiveness in the NHS context

PCM cooling mattresses were developed in a low-regulatory environment with clinicians testing the equipment clinically outside of trials or formally approved technology development processes [25]. This is characteristic of frugal innovation development processes [2]. Some of these products have received CE marking approval and ISO certification 13,485 [64], so barriers to scaling into the UK may be lower.

To the best of our knowledge, no trials have been carried out to test the effectiveness of PCM cooling mattresses for TH in HICs and this is important to develop interest in and appetite for this frugal technology. Evidence on the use of PCM cooling mattresses in the NHS context is necessary to support their adoption and to fully understand the compatibility of this innovation with the health system and how it would change current practice in rural or remote areas of the UK, since it may require more staff supervision compared to servo-controlled devices, including the need to monitor circulatory and respiratory parameters, and assess the requirement for sedation and respiratory support. A market analysis as well as interviews with professionals could further contribute to understanding the receptivity of the environment to this frugal innovation. Further, it may be valuable for PCM cooling mattresses to undergo a Health Technology Assessment (HTA) by the National Institute for Health and Care Excellence (NICE), to evaluate their cost utility with respect to other cooling devices.

##### Bias in reverse innovation adoption

A common challenge encountered in reverse innovation adoption is bias against products developed in low-resource settings, because of a dominating view favouring innovation from Western Europe and North America [3, 65]. This needs to be challenged, as the country of origin should not undermine the value of innovations in HIC healthcare settings. Whereas PCM technology was adapted for use in NIH in India, it is increasingly recognised for developing cost-effective healthcare innovations, doing more with less [66]. Like healthcare professionals, parents of neonates suffering from HIE also need to be convinced about the effectiveness of this device to treat HIE. This can be achieved with evidence from trials using PCM cooling mattresses, and by engaging with bodies such as the British Association of Perinatal Medicine or other charities and non-governmental organisations.

## Conclusion

PCM cooling mattresses offer a promising and cost-saving solution that should be considered for adoption by the NHS to ensure timely initiation of TH in remote areas of the UK where alternatives might not be readily available. This frugal innovation may help to reduce disparity in availability of treatment across the UK while meeting the challenges currently experienced by the NHS in terms of lack of funding and staff shortage. Further research is needed to provide evidence of effectiveness in the context of the NHS, as well as to understand changes in practice with respect to what is currently in use, in terms of supervision to monitor the device and amount of intensive care required. Moreover, further investigation into manufacturing and commercialisation of the product in the UK is needed, including a HTA by NICE. Lastly, bias against research and development from low-resource settings should not undermine the value behind such a promising innovation. These findings may be valuable for any HIC that experiences challenges in the provision of TH in geographically remote areas due to lack of cooling equipment.

## Data Availability

Not applicable.

## Abbreviations

HICs: High-income countries
HIE: Hypoxic ischaemic encephalopathy
HTA: Health technology assessment
LICs: Low-income countries
LMICs: Low- and middle-income countries
LNUs: Local neonatal units
NHS: National Health Service
NICE: National Institute for Health and Care Excellence
NICUs: Neonatal intensive care units
PCMs: Phase change materials
SCBUs: Special care baby units
TH: Therapeutic hypothermia
UK: United Kingdom
